# A comparative assessment of clinical efficiency between premium heat-activated copper nickel-titanium and superelastic nickel-titanium archwires during initial orthodontic alignment in adolescents: a randomized clinical trial

**DOI:** 10.1186/s40510-019-0299-4

**Published:** 2019-12-16

**Authors:** Ezgi Atik, Hande Gorucu-Coskuner, Bengisu Akarsu-Guven, Tulin Taner

**Affiliations:** Department of Orthodontics, Faculty of Dentistry, Hacettepe University, Sihhiye, 06100 Ankara, Turkey

**Keywords:** Alignment, Superelastic NiTi, Premium heat-activated Cu-NiTi

## Abstract

**Background:**

To compare the clinical efficiency of premium heat-activated copper nickel-titanium (Tanzo Cu-NiTi) and NT3 superelastic NiTi during initial orthodontic alignment.

**Subject and methods:**

A total of 50 patients were randomly allocated to 1 of 2 different archwire types (group 1, Tanzo Cu-NiTi; group 2, NT3 superelastic NiTi). Eligibility criteria included Class I or Class II malocclusion, moderate maxillary anterior crowding, and healthy periodontal condition. Impressions of the upper arches were taken before archwire placement (T0) and at every 4 weeks (T1, T2, T3, and T4). For T1 and T2 stages, 0.014-in., and for T3 and T4 stages, 0.018-in. archwires were used. The primary outcome was the alignment efficiency assessed using Little’s irregularity index. The secondary outcomes were arch width and incisor inclination changes. Data were analyzed using independent samples *t* test, repeated measures ANOVA, and Mann-Whitney *U* test. Marginal models were established for the estimation of coefficients.

**Results:**

The anterior irregularity index reduction was mostly observed between T0 and T2 periods, which were respectively − 7.40 ± 0.50 mm (*p* < 0.001; 95% CI, − 8.94, − 5.85) and − 6.80 ± 0.55 mm (*p* < 0.001; 95% CI, − 8.49, − 5.12) for groups 1 and 2 (*p* < 0.001). With both wires, Little’s irregularity index decreased over time, and the difference between the groups was not significant (*p* = 0.581; estimated effect size, 0.011). No statistically significant difference was found between the groups in terms of intercanine and intermolar width and incisor inclination changes.

**Conclusion:**

There were no significant between-group differences in alignment efficiency, arch width, and incisor inclination change. There was an increased alignment with 0.014-in. compared with 0.018-in. diameter archwire.

## Introduction

Nickel-titanium (NiTi) archwires are commonly used during the initial alignment of orthodontic treatment, since these wires have high elasticity and resilience with low elastic modulus and rigidity [1, 2].

As from the introduction of NiTi archwires into orthodontics, different elements have been added in order to provide clinical advantages. Copper is one of these elements that have been added to nickel-titanium, resulting in lowering the loading stress while providing relatively high unloading stress, which can result in more effective orthodontic tooth movement [3].

Heat-activated NiTi archwires have been introduced with clinically useful shape-memory, low stiffness, high spring-back, and superelasticity of the first and second generation NiTi archwires [4]. According to the scientific basis of the thermo-active wires [5], an archwire exerting a dynamic load might result to more desirable tissue reaction when compared with an archwire exerting a static load.

Several studies [6–8] found no significant difference in relation to alignment speed between different types of NiTi archwires. In two recent studies [9, 10], Cu-NiTi archwires were not found to be more efficient than NiTi archwires in leveling of mandibular anterior teeth. However, in contrast with the findings described above, one study [11] indicated more rapid correction of mandibular irregularity with heat-activated NiTi than NiTi archwires.

Tanzo Cu-NiTi archwire (Tanzo NiTi, American Orthodontics, Sheboygan, USA) is one of the newly developed premium heat-activated Cu-NiTi archwires. According to the manufacturers, addition of extra copper alloy into the wire allows lower loading and more consistent unloading forces, as copper content of Cu-NiTi enables the wires to apply more homogeneous forces, thus resulting more efficient tooth movement [12, 13]. However, whether using a premium heat-activated Cu-NiTi would result in better alignment efficiency when compared with superelastic NiTi archwires is still not clear especially for the maxillary anterior irregularity correction, since most of the studies in the literature were performed on mandibular arch. Besides, there is only one study [10] to compare the efficiency of this newly developed Cu-NiTi wire over the others.

In some systematic reviews [14, 15], it has been indicated that further parallel-randomized clinical trials should be conducted to justify the clinical superiority of any type of archwire. The aim of the present study was to evaluate the difference in the extent of maxillary anterior alignment between premium heat-activated Tanzo Cu-NiTi and superelastic NiTi (NT3 Superelastic NiTi, American Orthodontics, Sheboygan, USA) archwires by measuring the amount of tooth movement occurred at 4-, 8-, 12-, and 16-week intervals, and identify the confounding factors affecting improvement in anterior irregularity. The primary outcome measure of the trial was anterior alignment rate, whereas the secondary outcomes included maxillary arch width and incisor inclination change.

The null hypotheses of the study were that there is no difference between 2 different NiTi archwires in terms of maxillary anterior alignment efficiency, maxillary arch width change at the canine and molar regions, and incisor inclination change.

## Material and methods

The study was a single-center, 2-arm parallel-group, randomized with a 1:1 allocation ratio, and blinded prospective clinical trial. The study was approved by Hacettepe University Institutional Review Board with a number of KA-17005.

This was a single-center clinical trial, with three operators participating in the orthodontic treatment of the patients. The subjects were recruited from Hacettepe University, Faculty of Dentistry, Department of Orthodontics, and informed consent was obtained from the participants and their parent/guardian. The inclusion criteria were as follows: (1) patients with Class I or Class II malocclusion; (2) aged between 12 and 18 years with permanent dentition; (3) non-extraction treatment of the maxillary arch; (4) maxillary Little’s irregularity index of minimum 7 mm; (5) patients with no periodontal disease.

The patients were randomly allocated to 1 of 2 treatment groups, which were group 1 (premium heat-activated Tanzo Cu-NiTi) and group 2 (NT3 Superelastic NiTi).

Bracket bonding, archwire insertion, and following the treatment stages of the patients were performed by three experienced operators (E.A., H.G-C., B.A-G.) at the same clinic. The same fixed appliance 0.022 × 0.028-in. Roth prescription self-ligating brackets (Empower 2, American Orthodontics, Sheboygan, Wis) were bonded to the maxillary arch including the maxillary first molars. The archwire sequences for the groups were respectively; 0.014-in. and 0.018-in. premium heat-activated Cu-NiTi archwires (group 1), and 0.014-in. and 0.018-in. superelastic NiTi archwires (group 2).

**Fig. 1:**
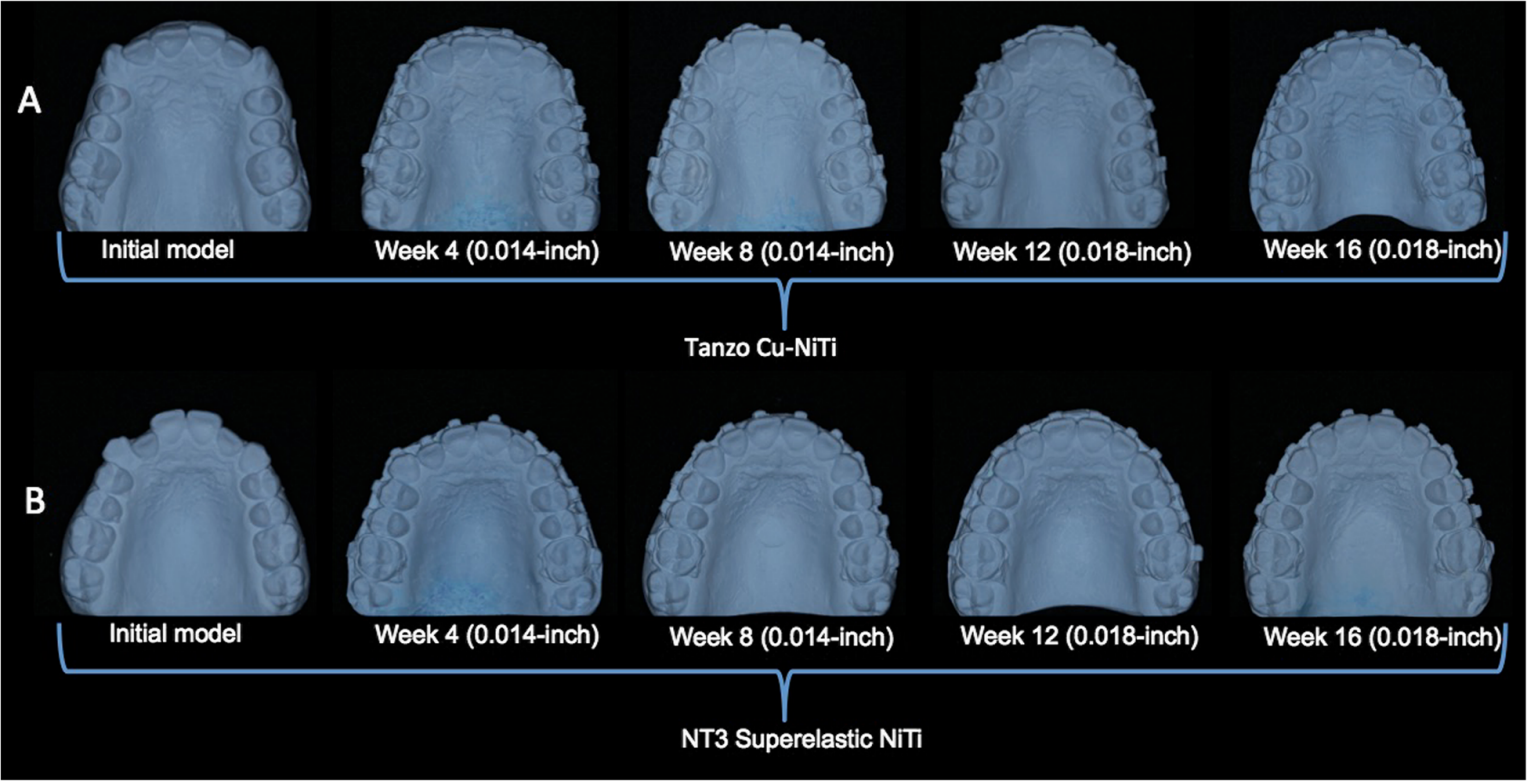
**a** Maxillary models of a patient for group 1. **b** Maxillary models of a patient for group 2

In both treatment groups, 0.014-in. archwires were kept in the mouth for 8 weeks, and thereafter 0.018-in. archwires were kept for 8 weeks. Both archwires presented natural arch form III (European arch form) as these forms were more compatible with patients’ maxillary dental arches. During the initial alignment stages, no auxiliary appliances were used. The patients were examined at 4-week intervals to ensure intact brackets. Maxillary impressions were taken first at the beginning of the treatment (T0) and thereafter at every 4 weeks (T1: at 4 weeks, T2: at 8 weeks, T3: at 12 weeks) up to 16 weeks of treatment (T4: at 16 weeks) (Fig. 1). At the 4- and 12-week appointments, the archwires were religated, without any modification to the wire, or to the brackets. The archwire was cut distal to the molar tube without cinching back.

The primary outcome was alignment efficiency, which was calculated by Little’s irregularity index. Also, arch width and incisor inclination changes were evaluated as secondary outcome measures in order to make a correlation between these mentioned parameters and alignment rate. All outcome measures were planned before the trial, and no changes were made after trial commencement.

Decreases of crowding between the treatment stages were measured in millimeters using Little’s irregularity index [16] by one operator. Measurements were taken of contact point displacements between canine and canine for maxillary models. Changes in Little’s index, intercanine (3-3), and intermolar widths (6-6) were assessed on plaster models using a digital caliper (Mitutoyo, Tokyo, Japan). Intercanine width measurements were made between cusp tips of canines, and intermolar width measurements were made between mesiobuccal cusp tips of first molars. Changes in incisor inclination were assessed using the following measurements on T0 and T4 lateral cephalograms: Mx1-SN (the angle of maxillary incisor to cranial base plane), Mx1-FH (the angle of maxillary incisor to Frankfurt Horizontal plane), Mx1-NA angle, and Mx1-NA distance (the angle and/or distance of maxillary incisor to Nasion-A point line).

The sample size calculation (G^*^ Power statistical software) indicated that with alpha risk of 0.05 and power of 95%, the study would require a minimum sample size of 21 patients in each group to distinguish a significant difference with in tooth movement between different archwire types (for one group, mean 4.1 mm, SD ± 0.7 mm; for the other group, mean 3.4 mm, SD ± 0.5 mm) according to the results of a study [17]. It was decided to select minimum 30 patients for each group with the expectation of drop-outs.

Randomization was used with block sizes of 4 to achieve a 1:1 allocation ratio. The archwire types were written and placed in a sealed opaque envelope for the allocation. This was performed before trial commencement by a secretary of the clinic, who was out of the study.

Operators were not blinded during the treatment period since it might be possible to discriminate the NiTi archwire types for the operators. Before the measurements, a person out of the study assigned an identification number for the study models in order to mask the patient name and the type of the archwire to make the researcher blinded during measurements. Besides, the name of the patients on cephalometric radiographs was blocked out. All dental cast and cephalometric measurements were carried out blindly by one investigator (E.A.). The same operator remeasured all variables of randomly selected 25 subjects 2 weeks after the first measurements to ensure intraexaminer reliability. Intraexaminer reliability was measured using both Bland-Altman plots and Intraclass Correlation Coefficient (ICC) analysis. The agreement of repeated measurements was within acceptable limits according to the Bland-Altman plots (Additional file 1: Figure S1 and Additional file 2: Figure S2).

### Statistical analysis

Data were analyzed using IBM SPSS Statistics version 22.0 (IBM, Armonk, NY). Data normality was checked via distributional diagrams and Shapiro-Wilk test. For the outcomes, which were not normally distributed, descriptive statistics were given as medians (25–75% percentiles). The other outcomes were presented as number (frequency %) or mean ± SD.

To compare the Little’s irregularity index, intercanine, and intermolar width changes between the different time appointments, Repeated Measures ANOVA with Bonferroni correction was used. To evaluate the intergroup differences, Greenhouse-Geisser results were used, as the Sphericity was not assumed. Mann-Whitney *U* test was conducted to evaluate and compare the incisor inclination changes. The level of statistical significance was set at *p* < 0.05.

To assess the influence of the gender, the diameter of the archwire, intercanine, and intermolar changes on the Little’s score changes, marginal models were established. For the estimation of coefficients, GEE (Generalized Estimating Equations) was used. The structure of correlation matrix in the model was based on exchangeable.

## Results

**Fig. 2:**
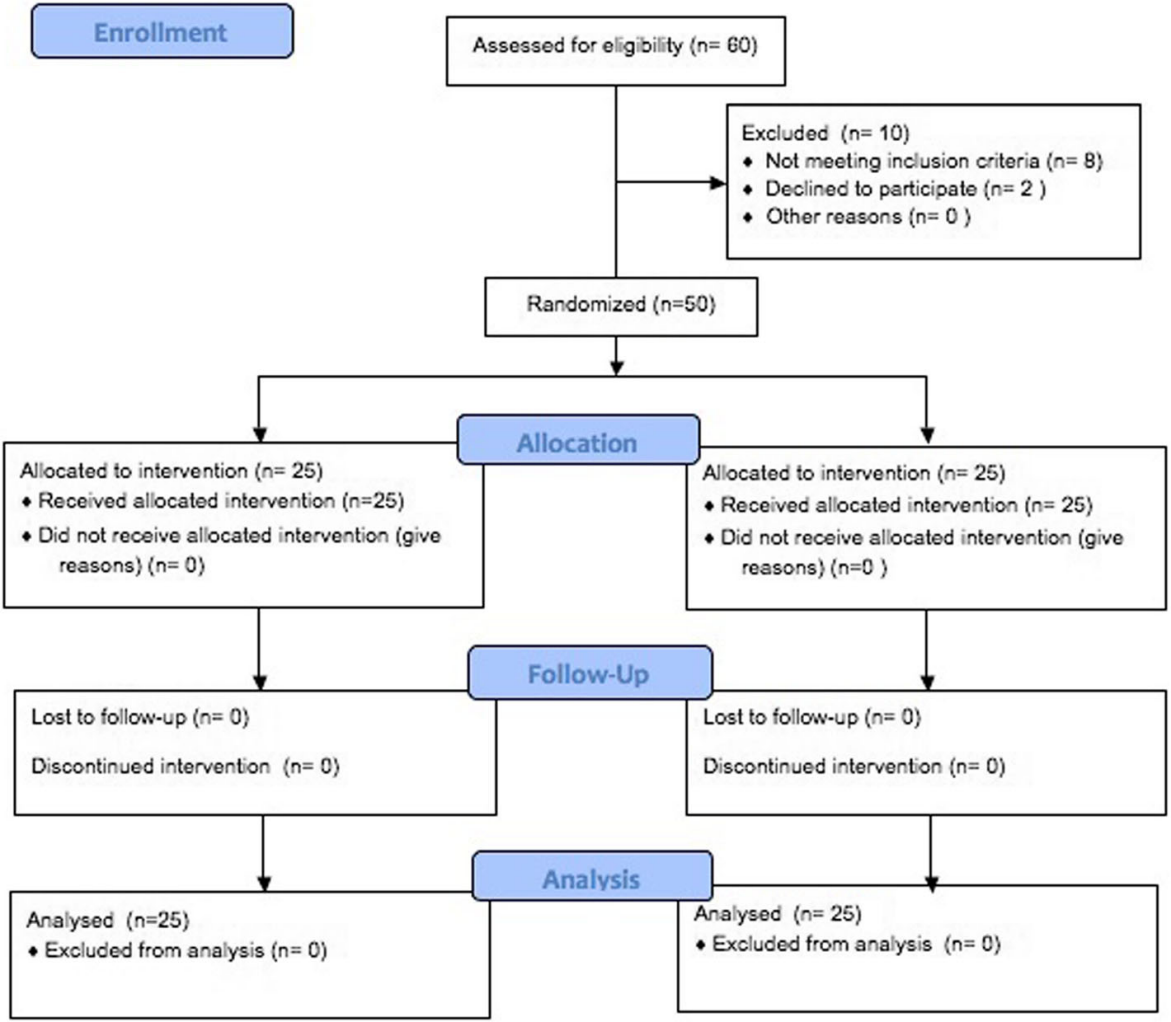
Consort flow diagram

Totally 60 potential subjects were assessed for eligibility; 8 of those were excluded due to not meeting the inclusion criteria. Two patients declined to participate to the research. Therefore, 50 patients were randomly allocated to either study (group 1, *n* = 25; 18 female; 7 male patients; mean age, 14.76 ± 1.77 years) or control group (group 2, *n* = 25; 19 female; 6 male; mean age, 14.75 ± 1.52 years) at the beginning of the trial, and all patients completed the trial. A CONSORT diagram showing the flow of the patients through the study is given in Fig. 2. The demographic and initial data on measurements for the groups are shown in Table 1. There were no significant differences between the groups with regard to age, gender, malocclusion type, and baseline values.

**Table 1:** Demographic distribution of subjects and baseline data for the groups

Variable	Group 1(heat-activated Cu-NiTi)	Group 2 (superelastic NiTi)	*p* value
Gender			
Female	18 (72%)	19 (76%)	1.000^a^
Male	7 (28%)	6 (24%)	
Malocclusion			
Class I	18 (72%)	12 (48%)	0.149^a^
Class II	7 (28%)	13 (52%)	
Age (years)	14.76 ± 1.77	14.75 ± 1.52	0.977^c^
ANB°	4.00 (2.95–4.65)	4.20 (2.65–6.45)	0.443^b^
Little T0 (mm)	9.95 ± 2.62	9.96 ± 2.77	0.986^c^
3-3 T0 (mm)	33.79 (31.40–34.66)	34.29 (32.85–36.04)	0.068^b^
6-6 T0 (mm)	49.48 ± 4.08	50.33 ± 2.97	0.401^c^
U1-SN° T0	102.70 (98.40–109.20)	105.90 (100.50–108.05)	0.415^b^
U1-FH° T0	111.48 ± 6.77	112.23 ± 5.94	0.678^c^
U1-NA° T0	18.40 (15.20–28.70)	21.70 (18.05–24.65)	0.946^b^
U1-NA T0 (mm)	4.20 (2.35–6.85)	4.30 (2.30–5.20)	0.655^b^

^a^Chi-square test

^b^Mann-Whitney *U* test

^c^Independent samples *t* test

Values are given as percentiles, mean ± SD, or median (25–75%)

Significant at *p* < 0.05

**Fig. 3:**
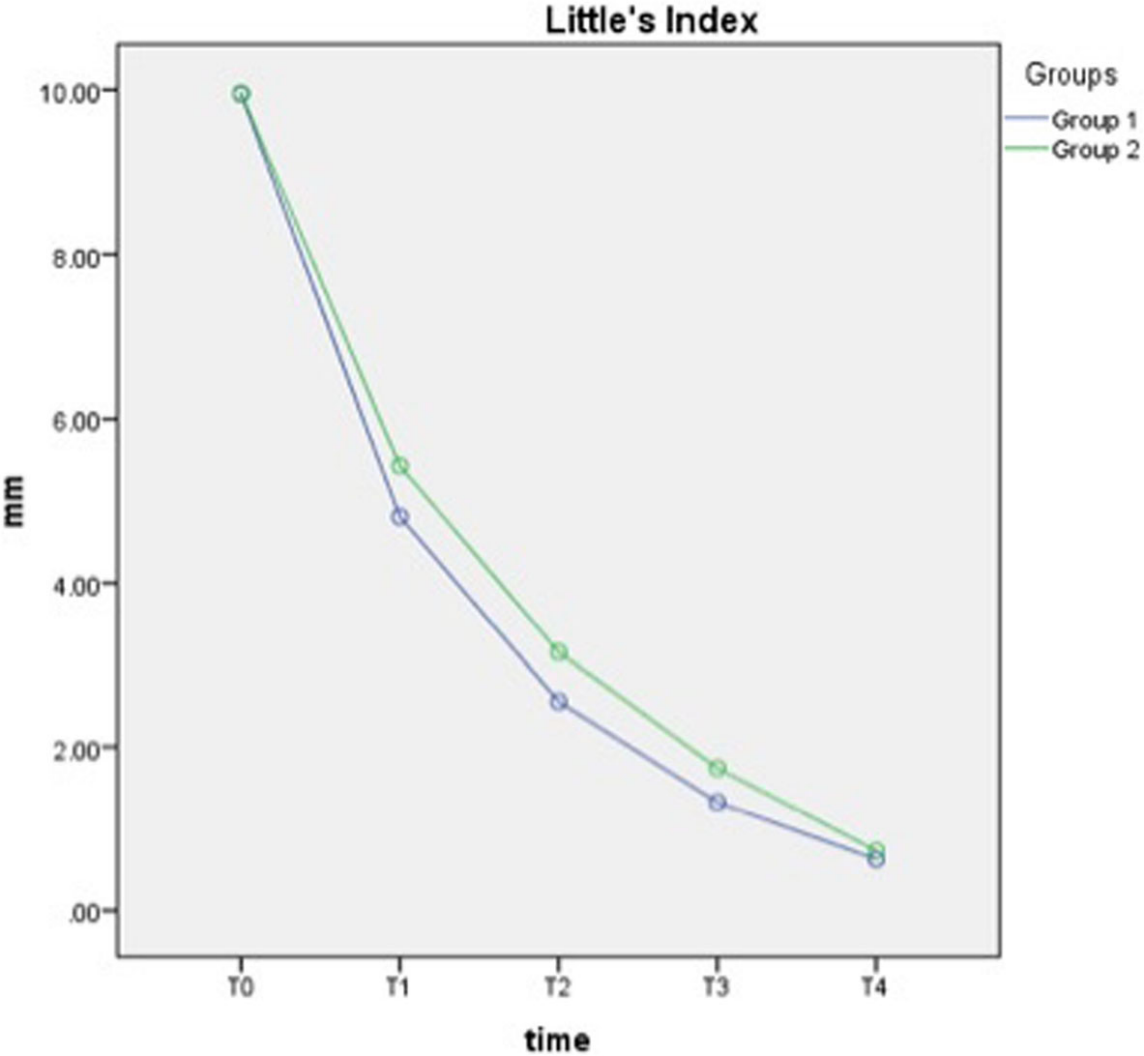
Change of the mean irregularity scores over time

The maxillary anterior irregularity reduction between T0 and T2 was − 7.40 ± 0.50 mm and − 6.80 ± 0.55 mm for groups 1 and 2 (*p* < 0.001), respectively. The anterior irregularity reductions between T2 and T4 were − 1.93 ± 0.21 mm and − 2.43 ± 0.24 mm for groups 1 and 2 (*p* < 0.001), respectively. With both types of wires, Little’s irregularity index decreased over time, and the difference in the reduction between the groups was not statistically significant (Table 2 and Fig. 3).

**Table 2:** Comparison of the differences in irregularity index between and within the groups

Little (mm)	Group 1 (heat-activated Cu-NiTi)				Group 2 (superelastic NiTi)				Time × group			
	Mean Diff. ± SE	95% CI		*p*^a^	Mean Diff. ± SE	95% CI		*p*^a^				
		Lower limit	Upper limit			Lower limit	Upper limit		Mean square	*F*	*p*^b^	Estimated effect size
T0-T1	− 5.14 ± 0.46	− 6.55	− 3.72	< 0.001	− 4.53 ± 0.47	− 5.98	− 3.08	< 0.001	2.028	0.538	0.581	0.011
T1-T2	− 2.26 ± 0.27	− 3.09	− 1.42	< 0.001	− 2.27 ± 0.34	− 3.32	− 1.23	< 0.001				
T2-T3	− 1.23 ± 0.17	− 1.74	− 0.71	< 0.001	− 1.42 ± 0.16	− 1.91	− 0.92	< 0.001				
T3-T4	− 0.70 ± 0.11	− 1.03	− 0.36	< 0.001	− 1.01 ± 0.14	− 1.44	− 0.59	< 0.001				
T0-T2	− 7.40 ± 0.50	− 8.94	− 5.85	< 0.001	− 6.80 ± 0.55	− 8.49	− 5.12	< 0.001				
T2-T4	− 1.93 ± 0.21	− 2.56	− 1.29	< 0.001	− 2.43 ± 0.24	− 3.18	− 1.68	< 0.001				
T0-T4	− 9.32 ± 0.50	− 10.85	− 7.79	< 0.001	− 9.23 ± 0.55	− 10.92	− 7.54	< 0.001				

Repeated measure ANOVA with Bonferroni correction was used to compare changes occurred with time

To evaluate the intergroup differences, Greenhouse-Geisser results were used, as the Sphericity was not assumed

*CI*, confidence interval; *SE*, standard error; *Est*., estimate

Significant at *p* < 0.05

*p*^a^, *p* value for the changes within the groups

*p*^b^, *p* value for the changes between the groups

**Fig. 4:**
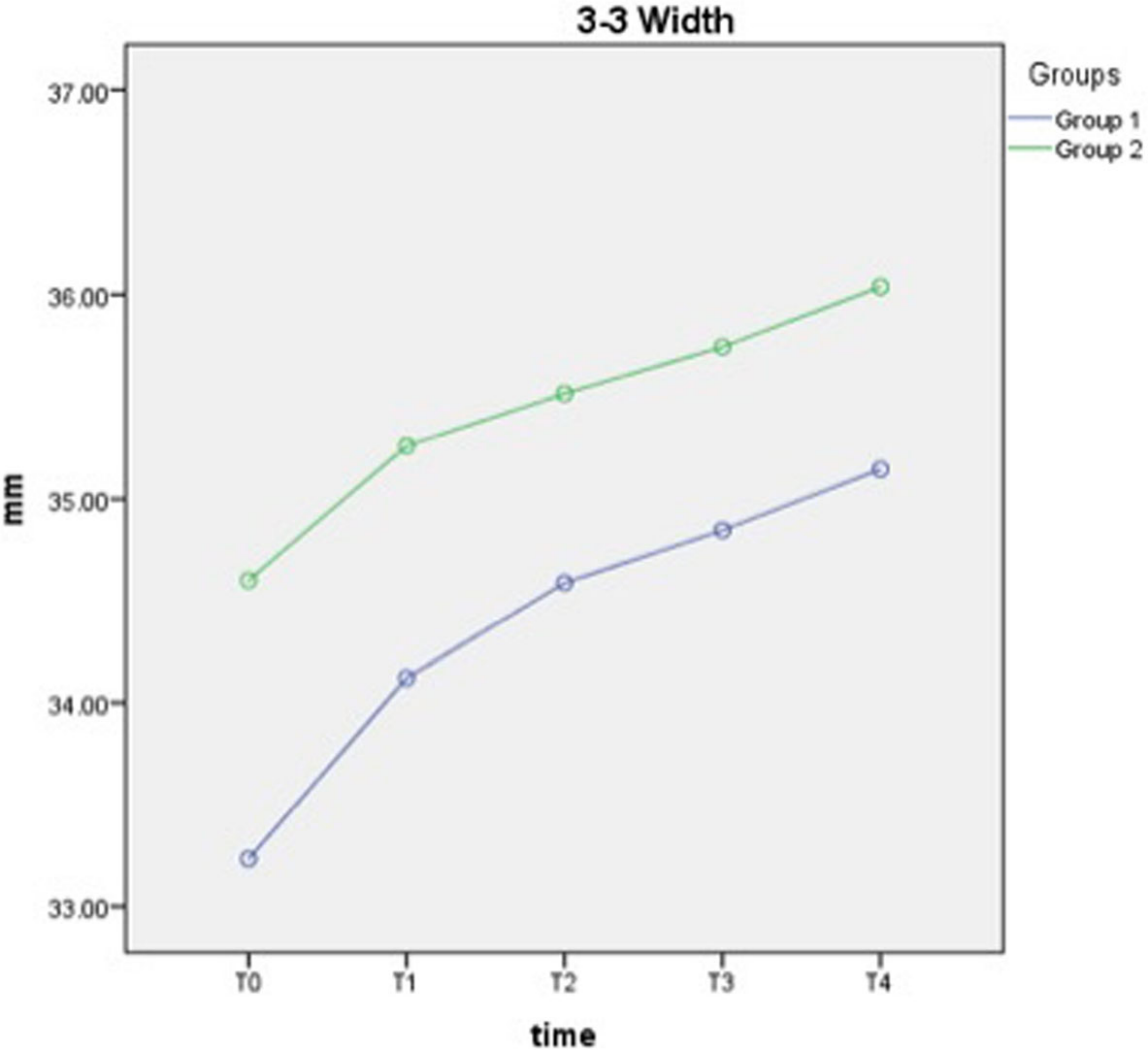
Change of intercanine width measurements over time

**Fig. 5:**
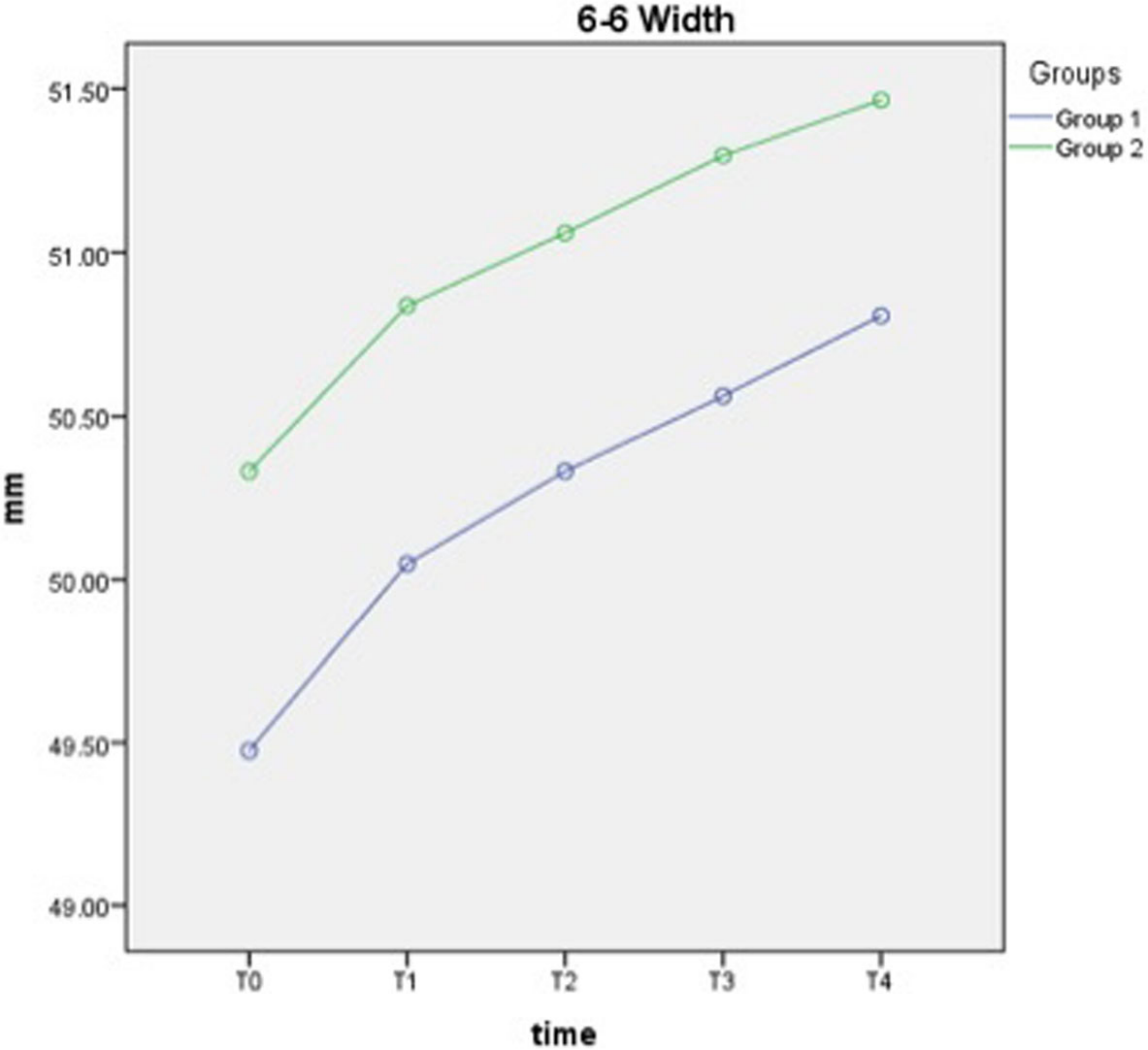
Change of intermolar width measurements over time

Total increases in transverse dimensions for intercanine from T0 to T4 were 1.91 ± 0.19 mm and 1.44 ± 0.23 mm for groups 1 and 2 (*p* < 0.001), respectively. Total increase for intermolar from T0 to T4 was 1.33 ± 0.14 and 1.13 ± 0.14 mm for groups 1 and 2 (*p* < 0.001), respectively. No significant difference was found between the groups in terms of intercanine and intermolar width changes (Table 3, Figs. 4 and 5).

**Table 3:** Comparison of intercanine and intermolar changes between and within the groups

	Group 1 (heat-activated Cu-NiTi)				Group 2 (superelastic NiTi)				Time × group			
	Mean Diff. ± SE	95% CI		*p*^a^	Mean Diff. ± SE	95% CI		*p*^a^	Mean square	*F*	*p*^b^	Estimated effect size
		Lower limit	Upper limit			Lower limit	Upper limit					
(3-3) Intercanine width (mm)												
T0-T1	0.89 ± 0.12	0.51	1.27	< 0.001	0.66 ± 0.15	0.21	1.11	0.001	1.178	1.909	0.158	0.038
T1-T2	0.47 ± 0.12	0.10	0.84	0.007	0.25 ± 0.09	0.02	0.53	0.082				
T2-T3	0.26 ± 0.07	0.05	0.46	0.007	0.23 ± 0.07	0.01	0.45	0.044				
T3-T4	0.30 ± 0.10	− 0.01	0.61	0.071	0.29 ± 0.09	0.01	0.58	0.040				
T0-T2	1.36 ± 0.19	0.77	1.94	< 0.001	0.91 ± 0.20	0.31	1.52	0.001				
T2-T4	0.56 ± 0.11	0.21	0.90	< 0.001	0.52 ± 0.11	0.18	0.87	0.001				
T0-T4	1.91 ± 0.19	1.32	2.51	< 0.001	1.44 ± 0.23	0.72	2.15	< 0.001				
(6-6) Intermolar width (mm)												
T0-T1	0.57 ± 0.12	0.21	0.94	0.001	0.51 ± 0.14	0.09	0.92	0.010	0.110	0.482	0.658	0.010
T1-T2	0.28 ± 0.06	0.11	0.46	0.001	0.22 ± 0.05	0.06	0.39	0.004				
T2-T3	0.23 ± 0.08	− 0.02	0.48	0.093	0.24 ± 0.05	0.07	0.40	0.002				
T3-T4	0.25 ± 0.08	0	0.49	0.054	0.17 ± 0.05	0	0.34	0.048				
T0-T2	0.86 ± 0.11	0.51	1.21	< 0.001	0.73 ± 0.14	0.30	1.16	< 0.001				
T2-T4	0.48 ± 0.11	0.15	0.80	0.002	0.41 ± 0.08	0.17	0.65	< 0.001				
T0-T4	1.33 ± 0.14	0.89	1.77	< 0.001	1.13 ± 0.14	0.69	1.57	< 0.001				

Repeated measure ANOVA with Bonferroni correction was used to compare changes occurred with time

To evaluate the intergroup differences, Greenhouse-Geisser results were used as the Sphericity was not assumed

*CI*, confidence interval; *SE*, standard error; *Est.*, estimate

Significant at *p* < 0.05

*p*^a^, *p* value for the changes within the groups

*p*^b^, *p* value for the changes between the groups

As shown in Table 4, the inclination change of maxillary incisors did not show significant difference except for U1-NA degree, which was significantly greater in group 2 than in group 1 (*p* = 0.044).

**Table 4:** Comparison of upper incisor inclination changes between the groups

		T0-T4 Mean Diff. ± SE	95% CI		*p*
			Lower limit	Upper Limit	
U1-SN°	Group 1	3.64 ± 0.65	2.29	4.99	0.479
	Group 2	4.54 ± 0.64	3.22	5.86	
U1-FH°	Group 1	3.64 ± 0.60	2.40	4.87	0.135
	Group 2	4.93 ± 0.62	3.66	6.20	
U1-NA°	Group 1	2.86 ± 0.58	1.66	4.06	0.044
	Group 2	4.70 ± 0.63	3.39	6.00	
U1-NA (mm)	Group 1	1.51 ± 0.32	0.85	2.17	0.662
	Group 2	1.85 ± 0.37	1.08	2.62	

Mann-Whitney *U* test was used for statistical significance

*CI*, confidence interval; *SE*, standard error

Significant at *p* < 0.05

When considering the effect of confounding variables on Little’s irregularity index change, the factor that significantly influenced was archwire diameter (Table 5). There was an increased alignment with 0.014-in. diameter (*p* < 0.001; estimate value, 3.60) according to the results of GEE models. Besides, intercanine width showed significant negative interaction (*p* = 0.048; estimate value, − 0.12) with irregularity change. GEE model did not reveal a significant effect for archwire group, gender, intermolar width change, group × archwire diameter, and group × time.

**Table 5:** Confounding variables for irregularity reduction, estimate, 95% confidence intervals, and estimate values for interactions

	Model 1			Model 2		
	Estimate	95% CI	*p* value	Estimate	95% CI	*p* value
Intercept	7.28		0.002	9.56		< 0.001
Group (superelastic NiTi/heat-activated Cu-NiTi)	1.10	(− 1.31, 3.51)	0.370	-	-	-
Diameter						
(No archwire/0.014)	4.01	(2.51, 5.51)	< 0.001	3.60	(2.50, 4.70)	< 0.001
(0.018/0.014)	0.24	(− 1.04, 1.51)	0.715	0.23	(− 0.64, 1.11)	0.597
Intercanine width	− 0.12	(− 0.25, − 0.00)	0.048	− 0.05	(− 0.06, 0.16)	0.367
Intermolar width	0.08	(− 0.43, 0.60)	0.055	-	-	-
Gender (female/male)	0.16	(− 0.35, 0.67)	0.537	-	-	-
Time	− 1.45	(− 2.05, − 0.85)	< 0.001	− 1.54	(− 1.96, − 1.13)	< 0.001
Group *×* diameter						
(Superelastic NiTi/heat-activated Cu-NiTi) *×* (no archwire/0.014)	− 0.82	(− 2.97, 1.33)	0.453	-	-	-
(Superelastic NiTi/heat-activated Cu-NiTi) *×* (0.018/0.014)	− 0.02	(− 1.73, 1.69)	0.982	-	-	-
Group *×* time						
(Superelastic NiTi/heat-activated Cu-NiTi) *×* time	− 0.17	(− 1.01, 0.66)	0.686	-	-	-

The same operator remeasured all variables of randomly selected 25 subjects 15 days after the first measurements. The range for Intraclass Correlation Coefficient (ICC) values was between 0.887 and 0.999. No serious harms were observed during the trial.

## Discussion

There are several trials [7–11, 18–20] in the literature to compare the alignment efficiency of different NiTi archwires, most of which are related to the leveling of the mandibular anterior teeth owing to the severity of crowding and reduced interbracket span. However, to the best to our knowledge, only few clinical studies [21–23] have studied the alignment efficiency of maxillary anterior teeth using different types of leveling archwires. So the present study focused on maxillary anterior alignment efficiency. Additionally, it was stated in a systematic review [15] that there was insufficient evidence whether there is a difference between thermoelastic Cu-NiTi and superelastic NiTi archwires, and it was emphasized that further well-designed randomized clinical trials were required. Consequently, in the present study, superelastic NiTi and premium heat-activated Cu-NiTi archwires were compared to determine the efficiency of maxillary anterior teeth alignment and the changes in intermaxillary arch dimensions by randomizing 50 patients. The possible factors such as age, gender, malocclusion type, crowding amount, bracket slot dimension, and interbracket span did not exhibit statistically significant difference between the groups, and this result may be thought to make our study stronger.

According to the results of the present study, there was no statistically significant difference between the archwire types in terms of irregularity reduction. However, there was a significant effect of archwire diameter on the amount of initial alignment according to the results of GEE models. The irregularity index mostly decreased during first 2 months (− 7.40 ± 0.50 mm for group 1 and − 6.80 ± 0.55 mm for group 2), in which period 0.014-in. archwires were used.

Abdelrahman et al. [8] compared the three types of 0.014-in. NiTi archwires in the mandibular arch up to the period of 16 weeks. Similar to the results of present study, superelastic and thermoelastic NiTi archwires did not differ from each other, and also from conventional NiTi in terms of alignment efficiency. Aydın et al. [9] compared natural arch form NiTi and Tru-Arch Cu-NiTi archwires in mandibular arch with a total duration of 12 weeks, and no significant difference was observed in terms of the alleviation of the crowding. The total crowding alleviation at the end of the periods was respectively 4.07 and 3.58 mm for NiTi and Cu-NiTi groups. Different from the studies [8, 9] mentioned above, the crowding alleviation amount found in the present study was greater, and 9.32 mm and 9.23 mm for respectively Cu-NiTi and Superelastic NiTi. The differences between the amounts of irregularity reduction of the present study and the studies’ mentioned above may arise from longer follow-up period, using 0.18-in. diameter at the second stage, and evaluating the maxillary arch instead of mandibular arch. In the maxillary arch, the increase of interbracket distance when compared with mandibular arch increases wire flexibility, resilience, and possibly the amount of tooth movement. Mahmoudzadeh et al. [18] compared the reduction of irregularity index in the mandibular arch from bonding to 4 weeks after, using either A-NiTi (superelastic) or heat-activated NiTi, and found no significant difference. Even the thermal NiTi wires exert significantly lower forces than superelastic wires of the same size, as indicated in the study of Gatto et al. [24], heat-activated NiTi showed the same clinical performance as 0.014-in. superelastic NiTi in the study of Mahmoudzadeh et al. [18]

Similar to the present study design, Gok et al. [10] evaluated Tanzo Cu-NiTi and conventional NiTi with 0.022-in. self-ligating brackets, and found no significant difference at 16 weeks in Little’s irregularity index scores. The main differences from our study were as follows: evaluating the mandibular arch, using 0.016-in. diameter at the second stage, and lower irregularity index compared with the present study, respectively.

In contrast to the results of the present study and the studies mentioned above [7–10, 18], Serafim et al. [11] concluded that heat-activated NiTi arch wires significantly alleviated the mandibular irregularity when compared with conventional NiTi archwires. However, the diameter of the archwires was changed monthly, and rectangular archwires were used at the third, fourth, and fifth months.

Throughout the alignment process, arch form changes occur in transverse dimensions, and these changes are necessary for alleviation of crowding in non-extraction cases [25, 26]. Considering the results of the present study, a gradual significant increase in arch dimensions from T0- to T2 and from T2- to T4 periods for both groups was noted. Nevertheless, the magnitude of expansion for intercanine and intermolar width was similar between the heat-activated Cu-NiTi and superelastic NiTi archwires using the same types of brackets in accordance with the results of other studies [9, 10]. As the forms of the archwires used in the present study were almost similar to each other, the obtained similar changes in arch widths can be thought as an expected result. This may be the case because the flexible 0.014 and 0.018 NiTi wires delivered only a little force because of a limited amount of observation time (4 months), and these wires were not capable of changing the arch width under these circumstances. Tanzo Cu-NiTi archwires also have “VLP type,” which presents wider arch form in the posterior segment compared with the Natural arch form used in the present study. Maybe with the use of these types of VLP Cu-NiTi archwires, different results would be obtained in the arch width.

The difference in alloy structure between the Cu-NiTi and conventional NiTi archwires [4] may result in different tendency with regard to the protrusion of the upper incisors. According to the results of the present study, the only significant difference in related to inclination variables was observed in U1-NA degree, which was greater in superelastic NiTi group than in Cu-NiTi. This result may not be considered clinically significant, as the other inclination variables did not show significant differences between the archwire types.

In low-friction mechanics, thermal wires are to be preferred to superelastic wires during the initial phases of alignment because of the ability of lower working forces at lower deflection levels [24]. Nevertheless, the structural differences between the two types of wires did not differ in clinical performance with regard to alignment rate and arch width changes in this study. Besides, the clinical impression by the authors was that heat-activated Cu-NiTi ascertained superior to the superelastic NiTi, since it was more easily engaged with especially grossly mal-aligned teeth.

The present study showed relatively short-term (0 to 16 weeks) outcomes. This can be considered as one of the limitations. Therefore, further prospective randomized clinical trials should be carried out to reveal the long-term effects of different types of NiTi archwires.

The trial was carried out only in one center, which was a university department. This can be considered as a limitation for the generalizability of our results. On the other hand, three operators were involved in the treatment process, and this could allow the findings to be applied more generally.

## Conclusions

1. There was no difference in maxillary anterior alignment efficiency between the superelastic NiTi and premium heat-activated Cu-NiTi archwires.
2. Superelastic NiTi and premium heat-activated Cu-NiTi archwires had similar effects in terms of changes in intercanine and intermolar width, and incisor inclinations.

## Supplementary information

**Additional file 1: Figure S1.** Bland-Altman plots for repeated measurements.

**Additional file 2. Figure S2.** Bland-Altman plots for repeated measurements.

## Data Availability

The data supporting the findings of this research can be obtained directly from the authors of the study.

## Abbreviations

Cu-NiTi: Copper nickel-titanium
GEE: Generalized Estimating Equations
NiTi: Nickel-titanium
