# Supplementary figures and images for: A comparative assessment of clinical efficiency between premium heat-activated copper nickel-titanium and superelastic nickel-titanium archwires during initial orthodontic alignment in adolescents: a randomized clinical trial
Source: Prog Orthod. 2019 Dec 16;20:46. doi: 10.1186/s40510-019-0299-4 (PMC6911816; doi:10.1186/s40510-019-0299-4)

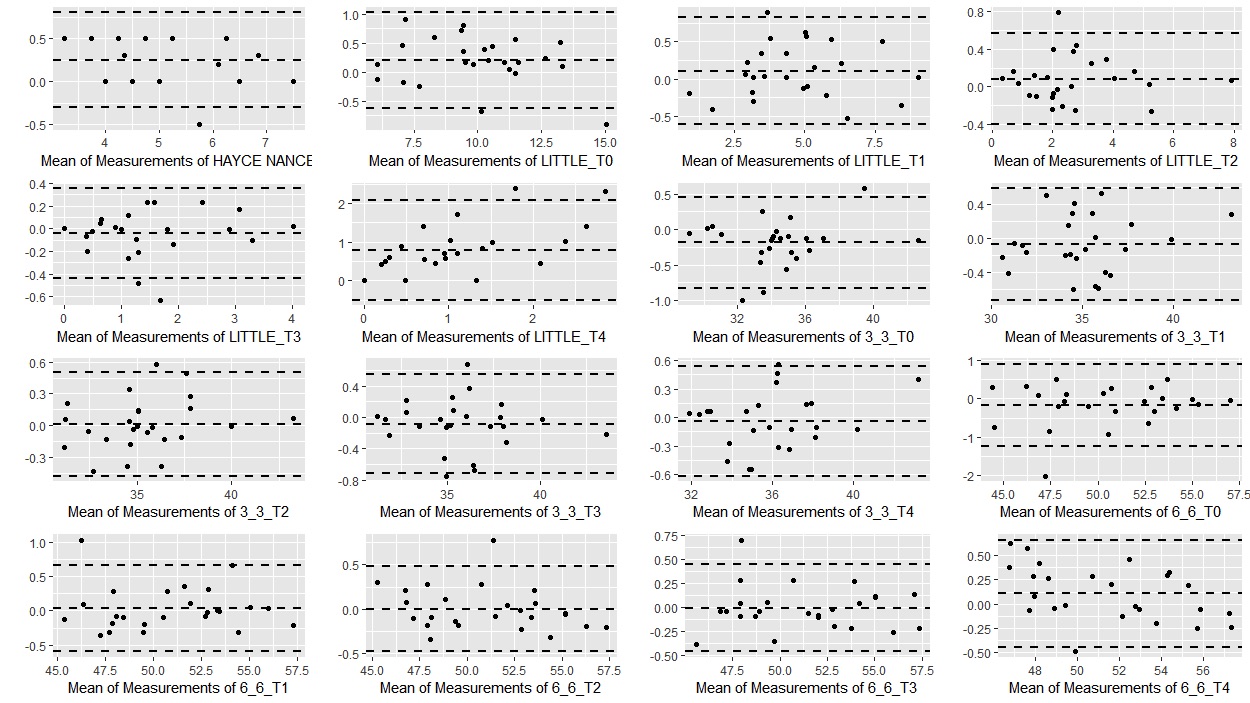

Supplement: Supplementary file 1 — Additional file 1: Figure S1. Bland-Altman plots for repeated measurements. [file 40510_2019_299_MOESM1_ESM.jpg]

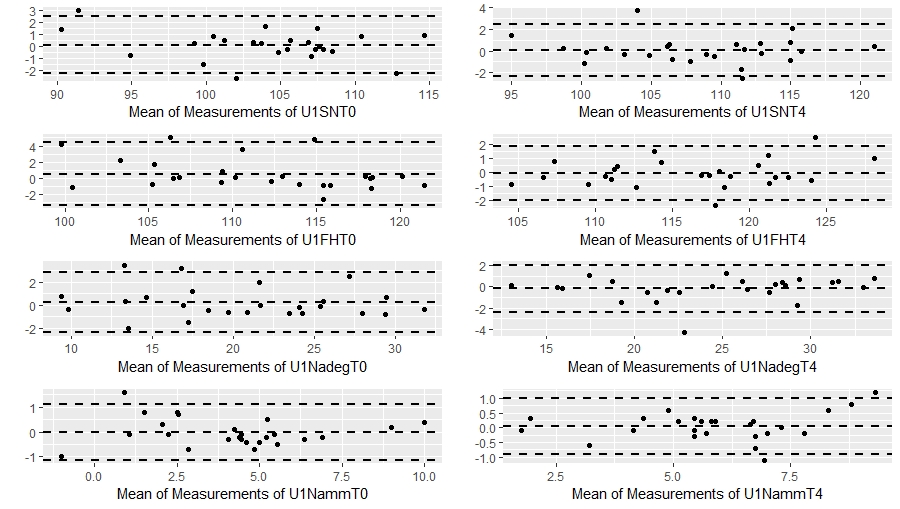

Supplement: Supplementary file 2 — Additional file 2. Figure S2. Bland-Altman plots for repeated measurements. [file 40510_2019_299_MOESM2_ESM.jpg]
